# The Mitogenomes and Phylogenetic Relationships of the Tribe Aedini in Culicidae (Diptera)

**DOI:** 10.1002/ece3.73734

**Published:** 2026-06-14

**Authors:** Wen‐Bo Fu, Huan Yuan, Hai‐Ju Ma, Xuan Dong, Bin Chen

**Affiliations:** ^1^ Chongqing Key Laboratory of Vector Control and Utilization; Institute of Entomology and Molecular Biology, College of Life Sciences Chongqing Normal University Chongqing China

**Keywords:** Aedini, characteristics, Culicidae, mitogenomes, phylogenetics

## Abstract

Aedini, the largest tribe within the Culicidae family, includes a large number of taxonomically complex assemblages. Despite decades of extensive research, their phylogeny remains unresolved owing to conflicting signals in traditional classifications and molecular data. In this study, we sequenced and annotated the mitogenomes sequences of 10 species from Aedini. Based on a comprehensive analysis of the mitogenomes from 52 species, we investigated the key features including gene composition, AT‐bias, length variation, codon usage bias, and phylogenetic relationships within the tribe. The findings reveal that the mitogenomic characteristics in Aedini are consistent with those of other species within the Culicidae family. However, the traditional genus *Aedes* displays a highly complex phylogenetic structure. Several genera, especially *Armigeres*, *Haemagogus*, and *Heizmannia*, are phylogenetically embedded within the traditional *Aedes* clade and form a closely related sister group, which highlighting the limitations of the traditional classification system. This study verified the rationality of elevating many subgenera or groups to genera rank of traditional *Aedes*, which better reflects Aedini evolutionary history, and provides a robust dataset for future taxonomic research. However, the relationship of its classification still needs to be further explored by adding more representative species.

## Graphical abstract

Aedini is the largest tribe in terms of species number and includes a large number of taxonomical complex assemblages in Culicidae. Its phylogeny and taxonomy have long been controversial and unresolved. This study endorses recent proposals to elevate many Aedes subgenera to generic rank, supports Harbach's revised generic taxonomic framework for its better reflection of Aedini evolutionary history, and provides a robust dataset for future taxonomic research on the tribe Aedini (Culicidae).

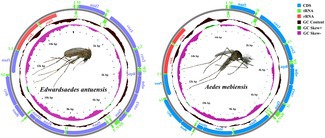

## 1 Introduction

The tribe Aedini (family Culicidae) is an important group of vector insects widely distributed throughout the world and serves as the primary vector for diseases such as dengue fever and chikungunya fever, especially through the mosquitoes *Stegomyia albopictus* and *Stegomyia aegypti* (Moyes et al. 2017; Gómez et al. 2022). It encompasses 1296 extant species, making it the largest tribe within the family Culicidae, and exhibits exceptional taxonomic complexity (Harbach 2024, 2025; Wilkerson et al. 2021). However, the fossil record for Aedini remains sparse despite this diversity. Notably, the most substantial increase in speciation rates within the Culicidae family—occurring approximately 20 to 30 million years ago—has significantly impacted the evolutionary history of Aedini lineages (Cockerell 1916; Szadziewski 1998). This period marks a major evolutionary divergence that has shaped the genetic and geographic distribution of mosquito species within this tribe.

Traditionally, Aedini has been classified into nine genera: *Aedes* (commonly referred to as traditional *Aedes*), *Armigeres*, *Eretmapodites*, *Haemagogus*, *Heizmannia*, *Opifex*, *Psorophora*, *Udaya*, and *Zeugnomyia*, and 52 subgenera (Knight and Stone 1977; Lu et al. 1997). Previously, *Verrallina* and *Ayurakitia* were considered subgenera of the traditional *Aedes*; Reinert elevated them to the genus rank within Aedini (Reinert 1999, 2000a). Subsequently, Reinert et al. classified the remaining species into two genera, *Aedes* and *Ochlerotatus*. Based on extensive morphological examinations of genitalia that covered over 65% of the currently recognized species and all subgenera in traditional *Aedes*, fourth‐instar larvae and pupae of the *Aedes* (Reinert 2000b). Following a series of studies (Reinert et al. 2004, 2006, 2008, 2009; Harbach 2007), the traditional *Aedes* was divided into 73 genera, with two groups remaining unclassified; consequently, the Aedini were divided into 84 genera and 123 subgenera (http://mosquito‐taxonomic‐inventory.info/) (Reinert et al. 2009; Harbach 2025), while there is another division into 10 genera and 92 subgenera by some others (Wilkerson et al. 2015, 2021; Harbach 2018, 2024). Although there is intense controversy over the taxonomy of Aedini, the formal proposal of the new combination originated around 2000 (Reinert 1999, 2000a). Since then, a series of academic works have supported and commented on it, and the International Commission on Nomenclature has also made targeted regulations (Qu and Zhu 2013; Weaves 2005; Marcondes 2007; Polaszek 2007).

Mitochondria possess maternally inherited compact circular genomes, which lack recombination and mutate at a relatively steady rate, and retain clear evolutionary signals across generations. Therefore, mitochondrial genomes have several advantageous features for evolutionary, phylogenetic, and population‐genetic studies, and their effectiveness has been demonstrated across numerous insect taxa (Cameron et al. 2007, 2009; Cameron 2014a; Fenn et al. 2008; Zadra et al. 2021; Wu et al. 2022). The assembly of mitochondrial genomes is labor‐intensive and expensive if first‐generation Sanger sequencing technology is used (Mitchell et al. 1993; Hua et al. 2016), with the advent of second‐ and third‐generation technologies, several efficient strategies—such as short‐read next‐generation sequencing (NGS) and long‐read shotgun genome sequencing from DNA‐seq libraries—now make mitogenome assembly routine (Briscoe et al. 2016; Cameron 2014b). In recent years, exploring the evolutionary history of mosquitoes, along with investigations into their systematic relationships, has utilized mitogenome sequencing and analysis (do Nascimento et al. 2021; Hao et al. 2017; da Silva, Machado, et al. 2020; Lorenz et al. 2021; Aragão et al. 2022; Chen et al. 2024; Guo et al. 2021; Ma et al. 2022). Additionally, mitogenomic studies have been conducted to elucidate phylogenetic relationships among species and genera in Aedini; for instance, four species in *Haemagogus* and 27 species in Aedini (da Silva, Cruz, et al. 2020; Zadra et al. 2021). However, due to insufficient data at both the species and genus levels, the phylogenetic and evolutionary relationships of the Aedini show inconsistencies between mitochondrial and nuclear gene analyses and still require more species data for further investigation.

In this study, we sequenced and annotated the mitogenomes of 10 species of the tribe Aedini, and compared them with 42 published Aedini mitochondrial genomes accessible via NCBI, along with two *Culex* species as outgroups. Phylogenetic analyses of all 52 mitogenomes to discuss the relationships within Aedini. It is findings that the taxonomic status of traditional *Aedes* and *Armigeres* is different from the traditional morphological understanding (Knight and Stone 1977; Lu et al. 1997). *Armigeres* and *Stegomyia* (it was a subgenus in traditional *Aedes*), important groups that pose a threat to human and animal health, have a closer genetic relationship. They may have similar vectorial capacity for disease transmission. These analyses also provide robust molecular evidence for systematic classification of the tribe, offering a foundation for future taxonomic research on Aedini.

## 2 Materials and Methods

### 2.1 Sampling Collection

In this study, 10 species of mosquito adults were collected, including *Aedes sasai*, *Aedes mebiensis*, *Armigeres subalbatus*, *Collessius elsiae*, *Downsiomyia albolateralis*, *Edwardsaedes antuensis*, *Hulecoeteomyia japonicus*, *Hulecoeteomyia koreicus*, *Ochlerotatus intrudens*, and *Stegomyia imitator* (detailed location information see Table S1), which represent new groups and distinct lineages of major species in Aedini. These samples were stored individually in 0.2 mL PCR tubes containing absolute ethanol or were dried and kept at −20°C. Voucher specimens were deposited at Chongqing Normal University (CNU).

### 2.2 DNA Extraction and Mitogenome Assembly

Genomic DNA was extracted from whole mosquito samples using the TIANamp Genomic DNA Kit (TIANGEN, Beijing, China), according to the manufacturer's instructions. DNA libraries were constructed with the Illumina TruSeq DNA Sample Preparation Kit (Illumina, San Diego, CA, USA) and sequenced on the Illumina Novaseq 6000 platform by Novogene (Beijing, China), generating 150 bp paired‐end reads. The mitochondrial genome was expected to be a circular molecule of approximately 15,000 bp, and the average sequencing depth was approximately 300×.

Raw reads were subjected to quality control and filtering, using the NGS QC toolkit (v.2.3) and Fastp (v.0.26.0) (Patel and Jain 2012). The resulting clean reads were used for de novo mitochondrial genome assembly with GetOrganelle v.1.7.7.1. using the “animal_mt” mode (Jin et al. 2020) and NOVOPlasty v.4.3.5 (Dierckxsens et al. 2017). The assemblies generated by the two programs were not directly merged; instead, they were compared and used for reciprocal validation. Final mitochondrial genome assemblies were determined based on concordance between the two assembly results and read‐level evidence, rather than on arbitrary merging of outputs from different assemblers.

For further validation of assembly accuracy, clean reads were mapped back to each circularized mitochondrial genome. Read mapping was performed using BWA, and the resulting alignments were processed with SAMtools to generate sorted BAM files and per‐base sequencing‐depth profiles (Jung and Han 2022; Li et al. 2009). The mapped reads were used to inspect base‐level accuracy, correct potential assembly errors, and assess whether the entire circular molecule was continuously supported by sequencing reads. Assembly reliability was evaluated according to the following criteria:

(i) Successful recovery of a circular mitochondrial genome;

(ii) Continuous read coverage across the entire molecule;

(iii) Absence of internal gaps or ambiguous bases;

(iv) Absence of abrupt coverage breaks or obvious low‐depth regions; and

(v) Read support across the junction region of the circular molecule.

### 2.3 Mitogenome Characteristics and Divergence Analysis

Assembled mitochondrial sequences were annotated using MitoZ (v.3.4) (Meng et al. 2019). Protein‐coding genes, ribosomal RNA genes, transfer RNA genes, and the putative control region were identified and manually checked. The locations of each gene were initially determined through the MITOS web server (http://mitos.bioinf.uni‐leipzig.de/index.py) (Bernt et al. 2013) and manually corrected in Geneious (v.9.2) (Kearse et al. 2012). Comparative analysis with available Culicidae mitogenomes in GenBank identified 13 protein‐coding genes (PCGs), two ribosomal RNA (rRNA) genes and 22 transfer RNA (tRNA). All sequences were corrected for low‐quality regions and frameshift mutations. Genomes were visualized using CGView (https://js.cgview.ca/examples/example‐mito.html) and Proksee (https://proksee.ca/) (Stothard and Wishart 2005; Grant et al. 2023). The annotated mitogenomes were deposited in GenBank under the Accession numbers PV191282–PV191283, PV191285–PV191291, and PV199093. Nucleotide composition was analyzed and assessed by AT/GC skew and content, relative synonymous codon usage (RSCU) and matrix of nucleotide distances built by Maximum Composite Likelihood were examined using software MEGA (12) and PhyloSuite (v.1.2.3) (Tamura et al. 2013; Zhang et al. 2020). The sequences were aligned using Clustal_X. Genetic divergence was calculated based on the PCGs using a 200 bp sliding window in DnaSP (v.5.10.01) (Librado and Rozas 2009). Because the A+T‐rich control region was incompletely or sparsely recovered in several taxa, subsequent comparative analyses focused on the PCGs, supplemented by the 22 tRNA and two rRNA genes.

### 2.4 Phylogenetic Analysis

Phylogenetic relationships were reconstructed using 52 mitogenomes from the tribe Aedini (including 10 newly sequenced mitogenomes from this study) and two species of genus *Culex* as outgroups (Table 1).

**TABLE 1:** Species in Aedini and their NCBI accession numbers of mitochondrial genomes analyzed in this study.

Genus	Subgenus	Specics	Accession no.	Note
*Aedes*	/	*cinereus*	PQ588179	
		*cinereus/geminus*	PV094688	
		*mebiensis*	PV191291	This study
		*rossicus*	PQ588180	
		*sasai*	PV191282	This study
*Aedimorphus*	/	*vexans*	NC065121	
*Armigeres*	*Armigeres*	*subalbatus*	PV199093	This study
		*subalbatus*	KY978578	
*Collessius*	*Collessius*	*elsiae*	PV191286	This study
*Dahliana*	/	*geniculata*	PV094711	
*Dobrotworskyius*	/	*alboannulatus*	NC054319	
		*rubrithorax*	MN389466	
*Downsiomyia*	/	*albolateralis*	PV191285	This study
*Edwardsaedes*	/	*antuensis*	PV191290	This study
*Georgecraigius*	*Horsfallius*	*fluviatilis*	OK662579	
*Haemagogus*	*Conopostegus*	*leucocelaenus*	MN531847	
	*Haemagogus*	*albomaculatus*	MN531846	
		*janthinomys*	KT372555	
		*spegazzinii*	MN531848	
		*tropicalis*	MN531849	
*Heizmannia*	*Mattinglyia*	*achaetae*	ON254136	
*Howardina*	/	*busckii*	MN626443	
*Hulecoeteomyia*	/	*japonica*	MZ566802	
		*japonica*	PV191288	This study
		*koreica*	PV191283	This study
		*koreica*	NC046946	
*Mucidus*	*Mucidus*	*alternans*	NC054325	
*Ochlerotatus*	*Culicelsa*	*taeniorhynchus*	MN626442	
	*Empihals*	*vigilax*	KP721463	
	*Ochlerotatus*	*scapularis*	NC072177	
		*trivittatus*	NC067621	
	*Protoculex*	*serratus*	NC072178	
	*Rusticoidus*	*rusticus*	PV094700	
	*Woodius*	*intrudens*	PV191289	This study
	Uncertain	*annulipes/cantans*	PV094697	
		*cantans*	PQ588172	
		*caspius*	NC068782	
		*communis*	PV094703	
		*detritus*	PV094732	
		*flavescens*	PV094735	
		*nigrithorax*	MN389467	
		*punctor*	PV094706	
		*sticticus*	PV094728	
*Psorophora*	*Janthinosoma*	*albipes*	OK662581	
		*ferox*	OK662582	
	*Psorophora*	*saeva*	MK575486	
*Rampamyia*	/	*notoscriptus*	KM676218	
*Stegomyia*	*Stegomyia*	*aegypti*	EU352212	
	Uncertain	*albopicta*	MK575475	
		*flavopictus*	MT501510	
		*galloisi*	MW465951	
		*imitator*	PV191287	This study
*Culex* (outgroup)	*Neoculex*	*fergusoni*	MN389458	
	*Culex*	*pipiens pallens*	KT851543	

Substitution saturation at each codon position of the 13 mitochondrial protein‐coding genes (PCGs) was assessed using the Iss index in Data Analysis in Molecular Biology and Evolution (DAMBE) (v.7.2.102) (Xia 2013). Phylogenetic analyses were conducted utilizing PhyloSuite (v.1.2.3) (Zhang et al. 2020; Xiang et al. 2023). Individual mitochondrial genes were aligned separately with Multiple Alignment using Fast Fourier Transform (MAFFT) (v.7.388) (Katoh and Standley 2013), and default parameters, followed by gap removal with trimAl (v.1.2rev57) (Capella‐Gutierrez et al. 2009) using the “‐automated1” command. Concatenation of aligned sequences was conducted in PhyloSuite (v.1.2.3). Maximum likelihood (ML) phylogenies were inferred using IQ‐TREE (v.2.2.0) (Nguyen et al. 2015). The best‐fit substitution model (GTR+F+I+I+R6) for both the 13 PCG genes dataset and the combined dataset of all 37 mitochondrial genes (PCG+rRNA+tRNA), as well as the best‐fit partition model for each of the 37 genes, was selected using ModelFinder (v.2.2.0) (Kalyaanamoorthy et al. 2017) based on 10,000 ultrafast (Minh et al. 2013) bootstraps, the approximate Bayes test (Anisimova et al. 2011), and the Shimodaira–Hasegawa–like approximate likelihood‐ratio test (Guindon et al. 2010). Bayesian Inference (BI) phylogenies were estimated using MrBayes (v.3.2.7a) (Ronquist et al. 2012), with ModelFinder (v.2.2.0) (Kalyaanamoorthy et al. 2017) used to select the best‐fit model under the BIC criterion. Best‐fit model (GTR+F+I+G4) for 13 PCG and 37 genes (PCG+rRNA+tRNA) and the best‐fit partition model for each gene of the 37 genes. Under the models runs of 2,000,000 generations were performed with sampling every 100 generations. The initial 25% of samples were discarded as burn‐in after convergence was confirmed (average standard deviation of split frequencies < 0.01). The input and output files related to the phylogenetic analyses can be downloaded from the Supporting data.

## 3 Results

### 3.1 Genome Structure and Nucleotide Composition

**FIGURE 1:**
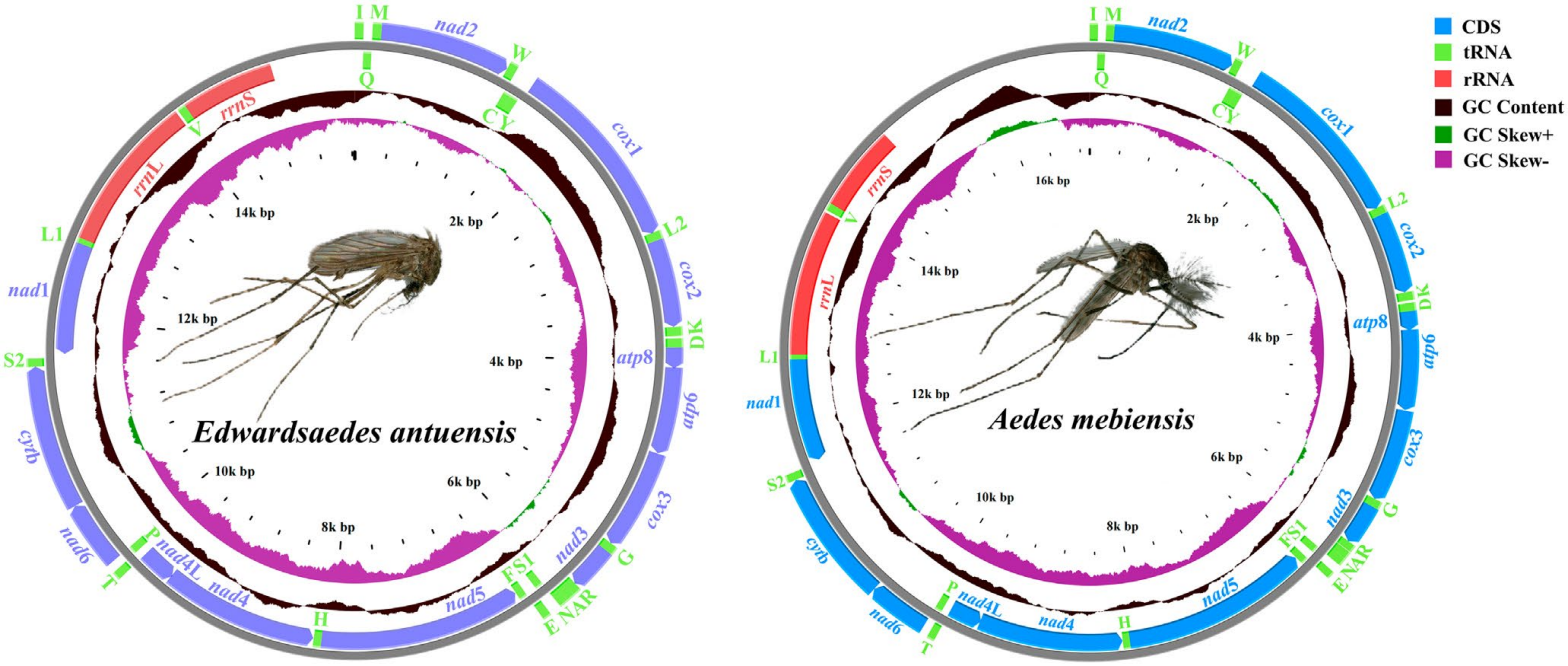
Circular maps of the complete mitochondrial genomes of the 2 representative species *Edwardsaedes antuensis* and *Aedes mebiensis*. Left is *Edwardsaedes antuensis* and right is *Aedes mebiensis*. The maps Contain genetic annotations, CG contents and CG skews.

Complete mitochondrial genomes were obtained for 10 Aedini species, providing the first mitogenomic records for three genera. Each mitogenome is approximately 16 kb in length and contains the standard 37 mitochondrial genes (13 protein‐coding genes, 22 tRNAs, and two rRNAs) along with an A+T‐rich control region. Nine PCGs and 13 tRNAs were encoded on the major strand (J‐strand), while the remaining genes were located on the minor strand (N‐strand) (Figure 1 and Figure S1). These patterns align with conserved characteristics observed across Culicidae and most insect mitogenomes (da Silva, Machado, et al. 2020; Lorenz et al. 2021; Aragão et al. 2022; Chen et al. 2024; Lemos et al. 2017; Sun et al. 2019; Yong et al. 2017; Dai et al. 2018).

Nucleotide composition analysis of the mitogenome sequences showed a remarkably strong A+T bias (Table S2), consistent with known characteristics of Culicidae. The A+T content of 37 genes and 13 protein‐coding genes (PCGs) ranges from 75.63% to 79.46%, and an average positive GC‐skew of −0.121 with a negative AT‐skew of 0.077. By contrast, the PCG subset displays negative AT‐skew (mean = −0.155) and positive GC‐skew (mean = +0.039). Synonymous codon usage analysis of protein‐coding genes (PCGs) revealed a strong A/U bias. The most frequent codon was UUA (Leu; mean RSCU = 5.01), followed by AGA (Arg; 2.94), UGA (Stop; 2.66), and UCU (Ser; 2.57). This codon‐usage pattern is conserved across all 10 Aedini mitogenomes (Table S3).

### 3.2 Rearrangement Events and Genetic Divergence

**FIGURE 2:**
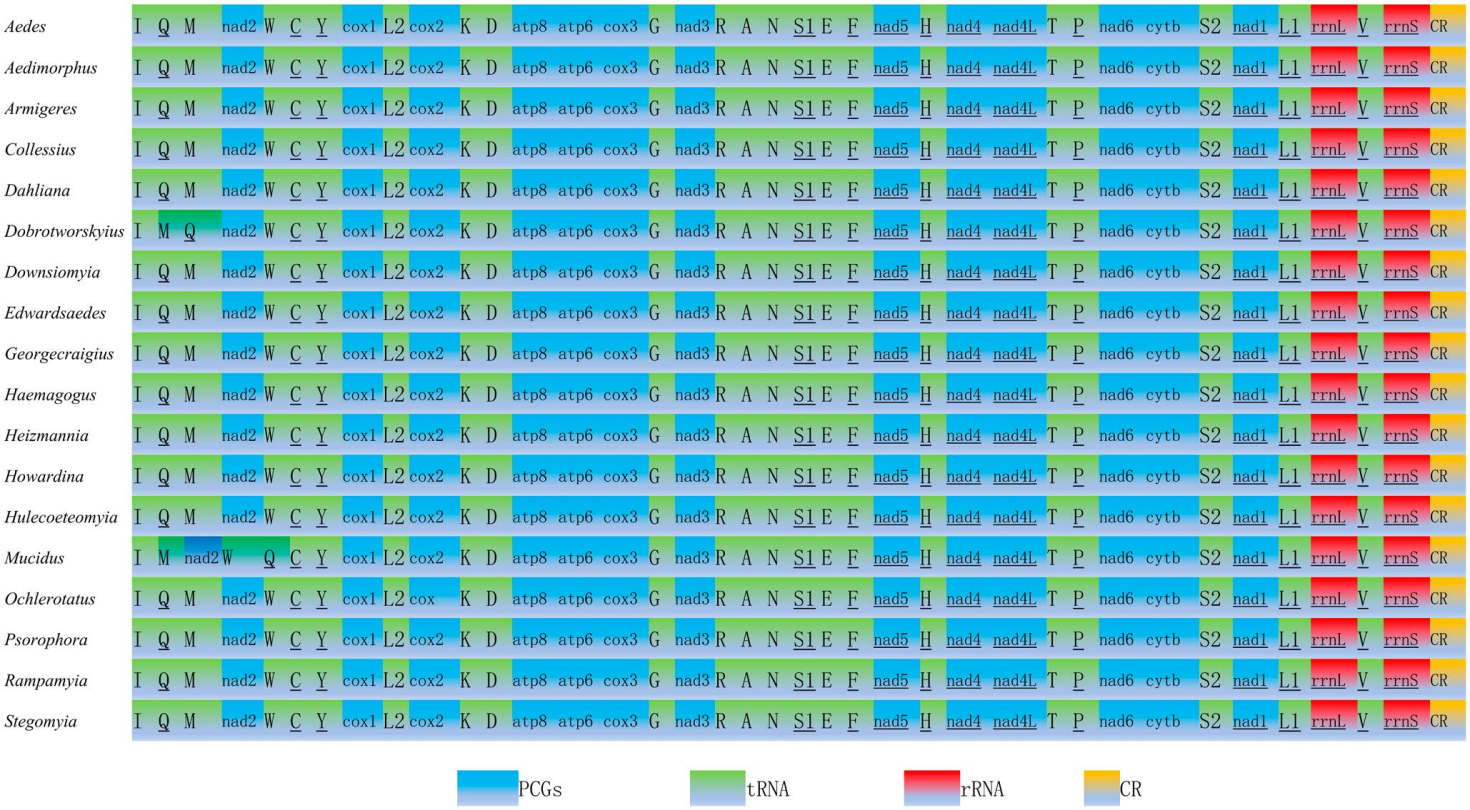
Gene rearrangement events in the 52 Aedini mt genomes. The underlined symbols are located on the N‐strand and others on the J‐strand. The green, blue, red and orange blocks denote PCGs, tRNAs, rRNAs and control regions, respectively. Thedeepen color means rearranged genes.

Comparative analysis of mitochondrial genome composition and structure across the 52 Aedini species revealed that mitochondrial gene order is identical to that of *Aedes* and is conserved across most Culicidae species. However, gene rearrangement events were identified in three species representing two genera: *Mucidus* and *Dobrotworskyius* (Figure 2). Rearrangements occurred most frequently in tRNA genes and notably in *trnI‐trnM‐trnQ* and *trnW‐trnC‐trnY* clusters. The *trnI‐trnM‐trnQ* cluster showed positional swaps of *trnM‐trnQ* in two *Dobrotworskyius* species, while protein‐coding genes remained largely conserved. The second event involves the transposition of the *trnQ* gene to the downstream region of *trnW* in the genus *Mucidus*. These species all belonged to the traditional genus *Aedes* before.

**FIGURE 3:**
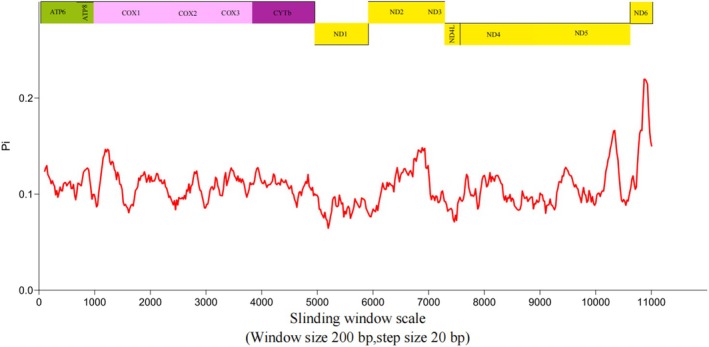
Genetic divergence among the mitogenomes of the 13 PCGs in this study. The size of the sliding window is 200 bp with each step of 20 bp. The bars above the graph represent protein‐coding genes (PCGs).

Sliding‐window analysis (200‐bp window) of the 13 PCGs across 52 Aedini mitogenomes revealed clear heterogeneity throughout the genome. Genome‐wide nucleotide diversity values spanned 0.064–0.220 within Aedini. The ND6 exhibited the highest divergence, followed by ND5, ND2, and COI (Figure 3).

### 3.3 Phylogenetic Relationships

In mitogenomes, the A+T‐rich control region has the characteristics of non‐coding genes and rapid evolution. However, insect phylogenetic analysis is rarely used alone due to its high AT repeatability, especially since the repetitive sequences in the control region of most Aedini mosquito groups are greater than those of other groups in the Culicidae, and no genetic function has been found in genetic studies (Wang et al. 2014). It is difficult to sequence accurately in the sequencing, especially in species such as *Armigeres*, where a large amount of genetic information is missing. Cutting off the A+T‐rich control region is more beneficial to the accuracy of phylogenetic analysis. Therefore, the genes in this region were not used for phylogenetic analysis in this study, but they can be explored in special topics in future research, especially in the study of related species.

**FIGURE 4:**
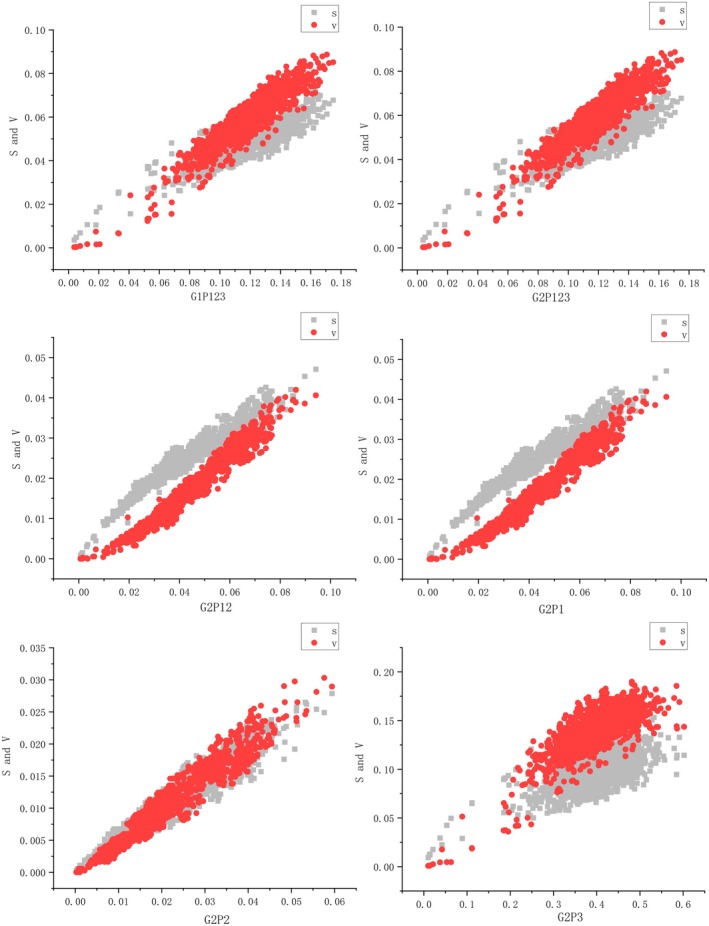
The chart of substitution saturation for the four different mitogenomes datasets. G1P123 comprises the 1st, 2nd, and 3rd codon positions of the complete sequences of all 37 genes, including 13 protein‐coding genes (PCGs), two ribosomal RNAs (rRNA) genes, and 22 transfer RNAs (tRNA) genes. G2P123 constitutes a set of the 1st, 2nd, and 3rd codon positions of 13 PCGs, with G2P12 denoting the 1st, and 2nd codon positions in this collection.

**FIGURE 5:**
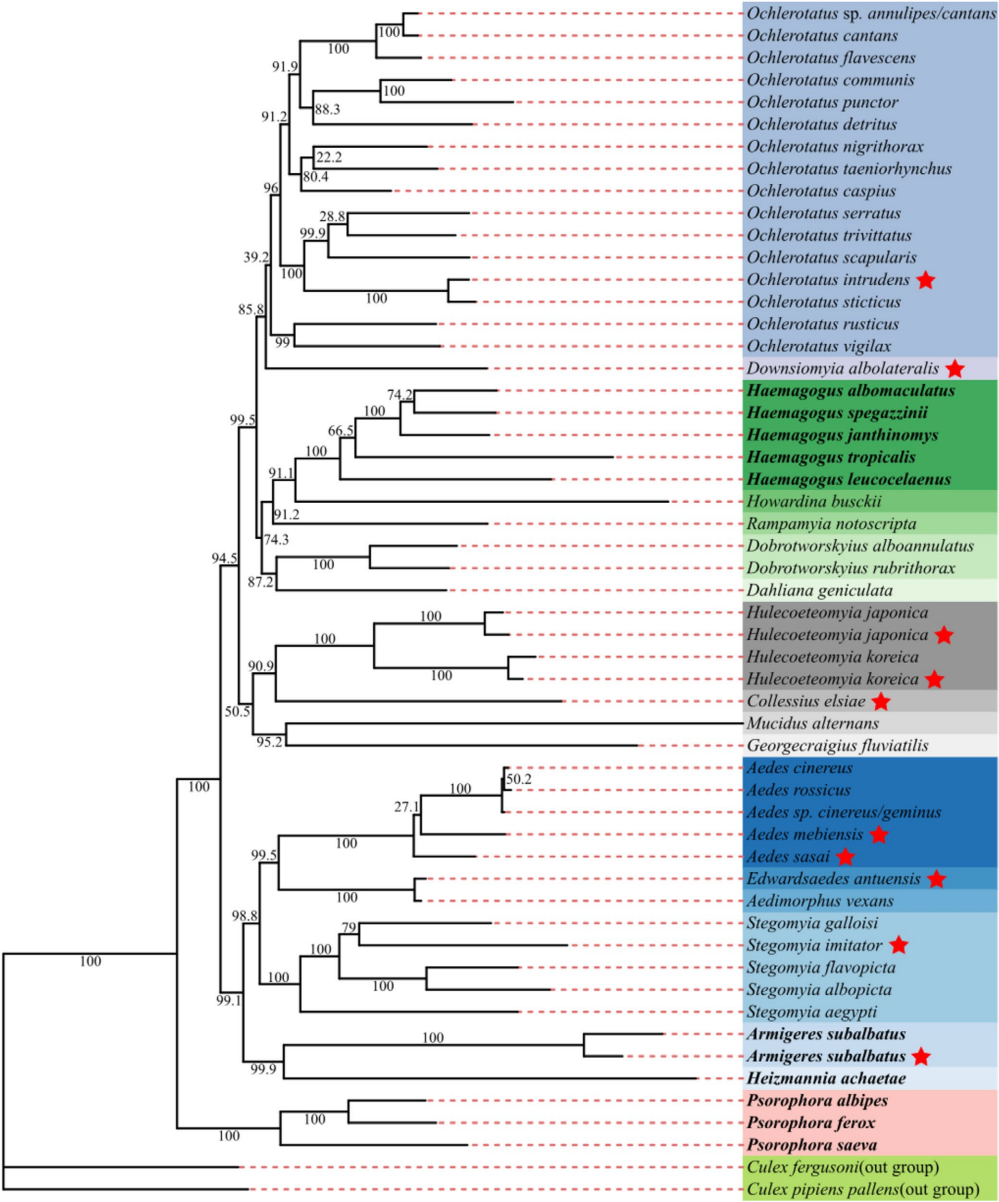
The phylogenetic tree generated based on the Maximum Likelihood methods (IQ) by concatenation of the 13 PCGs (The stars represent the species that were sequenced in this study).

Two concatenated matrices of the 52 mitogenomes—(G1) PCGs, 2 rRNAs, 22 tRNAs, and (G2) PCGs—were analyzed. Substitution‐saturation analysis confirmed minimal saturation in both datasets, indicating their reliability for phylogenetic reconstruction (Figure 4). Phylogenetic trees were constructed using maximum‐likelihood (ML) and Bayesian‐inference (BI) approaches. All six resulting phylogenies exhibited topological congruence at the genus level (Figure 5 and Figures S2–, S6). By comparing these six topological trees, the systematic relationships among genera are revealed as follows:

*Armigeres* and *Heizmannia* formed a polyphyletic group, which is a sister group to the genera of *Aedes*, *Aedimorphus*, *Edwardsaedes*, and *Stegomyia* that had belonged to the traditional *Aedes*. The phylogenetic relationships were ((*Armigeres* + *Heizmannia*) + ((*Aedes* + (*Aedimorphus* + *Edwardsaedes*)) + *Stegomyia*)), which formed a sister group to other genera in Aedini.

*Haemagogus* and the genera *Howardina* and *Rampamyia* which had belonged to the traditional *Aedes* constitute a polyphyletic group, the sister to the genera *Dobrotworskyius* and *Dahliana* (Figure 5). The phylogenetic relationships were (((*Haemagogus + Howardina*) *+ Rampamyia*) + (*Dobrotworskyius* + *Dahliana*)). *Ochlerotatus* forms a monophyletic group as a large cluster in this study. It has exhibited consistent relationships as a sister to *Downsiomyia* and other genera (*Haemagogus*, *Howardina*, *Rampamyia*, *Dobrotworskyius*, and *Dahliana*).

By analyzing the sister relationships across six phylogenetic trees, it was observed that *Rampamyia*, *Dobrotworskyius*, *Downsiomyia*, and *Dahliana* exhibited inconsistent evolutionary associations. They were swinging between the branches of *Ochlerotatus* and *Haemagogus*.

*Hulecoeteomyia*, *Collessius*, *Georgecraigius*, and *Mucidus* formed a sister clade. The phylogenetic relationships are ((*Hulecoeteomyia* + *Collessius*) + (*Georgecraigius* + *Mucidus*)). *Psorophora* is a monophyletic group as a sister group with the above clades in Aedini.

## 4 Discussion

The taxonomic relationships of the tribe Aedini are complex: however, the scarcity of existing mitochondrial molecular data impedes their clarification. Many generic taxa still lack relevant nucleotide sequence information. In this study, the authors collected samples of the tribe Aedini and sequenced 10 species, which supplemented the molecular data for this tribe.

Analysis of the sequenced mitochondrial genomes indicates conserved molecular characteristics (GC content, GC skew) and gene organization (composition, arrangement order) within the tribe Aedini, consistent across the family Culicidae (da Silva, Machado, et al. 2020; da Silva, Cruz, et al. 2020; Lorenz et al. 2021; Aragão et al. 2022; Chen et al. 2024). In codon usage analysis, the UUA (RSCU) was significantly higher than that of other codons at the species level and on average across the Culicidae species. However, other codons exhibited only minor variations across different species (Chen et al. 2024; Aragão et al. 2022). This may involve differences between species.

Mitochondrial genome rearrangements occur in select species of *Mucidus* and *Dobrotworskyius*, with tRNA genes being the most frequent sites of rearrangement. These rearrangement phenomena are more obvious in the higher classification order (Wang et al. 2014). These events may be mediated by aberrant tRNA‐primed mitochondrial replication or by tandem duplication events, thereby providing informative characters for phylogenetic inference (Cameron 2014a; Boore et al. 1998). The heterogeneity of PCGs reflects the conservation of different genes, which can be suitable for the phylogenetic analysis of the corresponding taxonomic order. These results of sliding‐window analysis are in line with previous studies and also indicate that these PCGs have a steady rate of genetic divergence, suggesting that they are suitable for evolutionary analysis.

Six phylogenetic trees were constructed, and although some species showed relatively low support, the internal relationships among their species were generally congruent across the topologies, resolving into five stable internal clusters. The phylogenetic topologies demonstrate that the genera of Aedini were difficult to distinguish at the traditional genus‐level. A key finding is the complex interrelationship exhibited by the traditional genus *Aedes*; some genera within Aedini show close sister‐group relationships with specific subgenera which is the traditional *Aedes*. For instance:

The clade comprises the genera *Aedimorphus*, *Edwardsaedes*, and *Aedes*, with *Stegomyia* formed a close sister‐group relationship. This clade also includes the genera *Armigeres* and *Heizmannia*. Notably, species in this cluster usually have similar morphological characteristics: The pedicel of their antennae are covered with scales, and their bodies are mainly black, with most individuals possessing broad and bright scales on their bodies. Based on morphological characteristics of adult mosquito genitalia and other features, Lu et al. (1997) previously proposed elevating *Armigeres* to the status of a separate tribe Armigerini. However, phylogenetic findings indicate that this genus does not warrant recognition as a distinct tribe. This conclusion aligns with the current international taxonomic framework (Harbach 2024; Wilkerson et al. 2021). Focusing on genus *Aedes*, the *Ae. mebiensis* is the sole species distributed in the Oriental region rather than the Palaearctic region. The genus *Stegomyia*, represents a large and diverse group of mosquitoes that are crucial vectors for the transmission of the yellow fever virus. Among the 133 species in the genus, only 32 have been assigned to subgenus‐level taxonomic groups (Harbach 2025). The *St. aegypti* is situated at the periphery of the branch, akin to the organizational framework for classifications. Situated at the edge of the branch, it aligns with its current classification as a separate subgenus within the morphological category.

The genus *Ochlerotatus*, as a larger cluster, is the most complex group within the Aedini. Currently, 75 species are grouped in thirteen subgenera, while 129 species are without subgeneric placement. In *Ochlerotatus*, the phylogenetic relationships are relatively stable, except that the relationships of the species *Oc*. *Taeniorhynchus*, *Oc*. *caspius*, and *Oc. nigrithorax* are different in the six trees. The species *Oc*. *intrudens* and *Oc. sticticus* formed a stable branch and belong to the same subgenus‐level group under *Woodius*.

*Haemagogus* forms a sister‐group with the genus *Howardina* which historically was part of the traditional *Aedes*. In contrast to the unstable topological positions of the genera *Rampamyia*, *Dobrotworskyius*, *Dahliana*, and *Downsiomyia* across six phylogenetic trees of two‐gene data and three different models. This instability may be due to the data composition, analytical methods and evolutionary models used for tree inference. By comparing the six topological trees formed by two datasets*—*13 PCGs genes and all 37 genes (single model and partition model)—and two computational methods (ML and BI), it is found that different datasets and methods have an impact on the research results, and the influence of dataset changes was greater than that of computational methods. In some genera, these mosquito groups exhibit significant differences in morphological characteristics and geographical distribution, such as *Rampamyia* (in the Australasia Region), *Dobrotworskyius* (in Australia), *Downsiomyia* (in the Oriental Region and adjoining areas of the Australasian and Palaearctic Regions), and *Dahliana* (in the Palaearctic Region) (Wilkerson et al. 2021). They exhibited unstable phylogenetic positions and low support values in the phylogenetic tree, which may be attributed to limited taxon sampling and to gene flow resulting from their geographic differentiation. This result may also be related to the insufficiency of species data and the taxonomic status of these genera requires further investigation.

## 5 Conclusions

This study examined the phylogenetic relationship within the tribe Aedini using all available species sequences, and this result is better supported than the results based on individual gene segments like COI (Wang et al. 2019). The classification of the traditional genus *Aedes* presents a highly complex and contradictory structure, with significant conflicts existing among the genus‐level taxonomic relationships in Aedini. Given that some genera, such as *Armigeres* and *Heizmannia* belong to the Aedini and it is more closely related to the *Stegomyia*, and for *Ochlerotatus* and *Stegomyia*, the genera's taxonomic status is consistent with molecular data, the subgenera's classification system relationships still require more data to clarify. Future research will require substantially more species data and nuclear genome data to address these research gaps.

This study verified the rationality of elevating many subgenera or groups to genera rank of traditional *Aedes* (Reinert et al. 2009; Harbach 2025), establishing a consistent framework for Culicidae taxonomy with enhanced resolution of relationships within the Aedini tribe. However, this classification also has a significant limitation: It generates excessive taxonomic ranks, and many species remain unassigned at the genus or subgenus level, so expanding more datasets representing species is crucial for determining the relationships among these systems. Nevertheless, these research methods are also applicable to the family Culicidae and other similar insects.

## Author Contributions

**Wen‐Bo Fu:** conceptualization (equal), methodology (equal), software (equal), supervision (equal), validation (equal), writing – original draft (equal). **Huan Yuan:** data curation (equal), investigation (equal). **Hai‐Ju Ma:** investigation (equal), software (equal). **Xuan Dong:** data curation (equal), investigation (equal). **Bin Chen:** conceptualization (equal), investigation (equal), methodology (equal), project administration (equal), resources (equal), supervision (equal), validation (equal), writing – review and editing (equal).

## Funding

This work was supported by Key Project of Chongqing Technological Innovation and Application Development Special Project (Project No.: CSTB2024TIAD‐KPX0085); Major Project of Science and Technology Research of Chongqing Municipal Education Commission (Project No.: KJZD‐M202100502); Science and Technology Research Program of Chongqing Municipal Education Commission (Project No.: KJQN202000504; KJQN202200566).

## Conflicts of Interest

The authors declare no conflicts of interest.

## Supporting information


**Figure S1:** Linear map of the 10 mitochondrial genomes in this study.

**Figure S2:** The phylogenetic tree generated based on the Bayesian Inference methods (BI) by concatenation of the PCGs.

**Figure S3:** The phylogenetic tree generated based on the Maximum Likelihood methods (IQ) by concatenation of the 37 genes PCGs+tRNAs+rRNAs.

**Figure S4:** The phylogenetic tree generated based on the Bayesian Inference methods (BI) by concatenation of the 37 genes PCGs+tRNAs+rRNAs.

**Figure S5:** The phylogenetic tree generated based on the Maximum Likelihood methods (IQ) under partition model of the 37 genes PCGs+tRNAs+rRNAs (GTR+F+I+I+R4 for atp6+cox3, TIM2+F+I+I+R4 for atp8+nad2, GTR+F+I+I+R4 for cox2+cytb+nad3+S2, TPM2u+F+R3 for L2+A+C+D+F+G+I+K+L1+L2+L+M+N+P+Q+R+S2+T+W+Y, GTR+F+I+I+R4 for nad1+nad4L+nad4+nad5, GTR+F+R4 for nad6+E, GTR+F+R3 for rrnL+rrnS+H+S1+S+V).

**Figure S6:** The phylogenetic tree generated based on the Bayesian Inference methods (BI) under partition model of the 37 genes PCGs+tRNAs+rRNAs (GTR+F+I+G4 for atp6+cox1, GTR+F+I+G4 for atp8+nad2, GTR+F+I+G4 for cox2+cox3+cytb+nad3, GTR+F+I+G4 for L2+S2+A+C+F+G+I+L1+L2+L+P+Q+R+S2+W+Y, GTR+F+I+G4 for nad1+nad4L+nad4+nad5, GTR+F+I+G4 for nad6, GTR+F+I+G4 for rrnL+H+K+M, GTR+F+G4 for rrnS+S1+S+V, GTR+F+I+G4 for D+E+N+T).

**Table S1:** Sampling collection information of the 10 species in this study.
**Table S2:** Composition and skewness of sequenced in this study. Composition and skewness in 37 genes (13 protein‐coding genes, 22 tRNAs, two rRNAs) and concatenated PCGs.
**Table S3:** The codon usage in contents and RSCU of 13 PCGs in the 10 species.

## Data Availability

The data that support the findings of this study are available in the Supporting Information. The read data reported in this study were submitted to the GenBank of NCBI numbers for PV191282–PV191283, PV191285–PV191291, and PV199093.
